# Long-Term Efficacy of Internet-Based Cognitive Behavioral Therapy Self-Help Programs for Adults With Depression: Systematic Review and Meta-Analysis of Randomized Controlled Trials

**DOI:** 10.2196/46925

**Published:** 2023-08-22

**Authors:** Megi Mamukashvili-Delau, Nicole Koburger, Sandra Dietrich, Christine Rummel-Kluge

**Affiliations:** 1 Department of Psychiatry and Psychotherapy Medical Faculty Leipzig University Leipzig Germany; 2 Department of Psychiatry and Psychotherapy Universitätsklinikum Leipzig Leipzig Germany; 3 Department of Research and Transfer Leipzig University Leipzig Germany; 4 Leipzig Travel Leipzig Tourismus und Marketing GmbH Leipzig Germany

**Keywords:** depression, internet-based cognitive behavioral therapy, iCBT, self-help, minimal guidance, long-term, follow-up, mental health, psychotherapy, cognitive behavioral therapy, CBT, systematic review, meta-analysis, meta-analyses, review method

## Abstract

**Background:**

Depression is a worldwide mental disorder and a leading cause of disability. Many people with depression do not want to take medication or have the motivation to seek psychotherapy treatment for many reasons. Guided internet-based self-help programs may be a promising solution for addressing these issues. This kind of intervention has proven to be effective in reducing depression symptoms on a short-term scale. However, as treatment often is a long-term rehabilitation process, it is important to examine not only the short-term effects of internet-based cognitive behavioral therapy (iCBT) self-help treatment but also the follow-up or long-term efficacy of this kind of intervention.

**Objective:**

This systematic review and meta-analysis aimed to identify studies that examined follow-up data ≥8 weeks after posttreatment measurements and thereby examined the long-term efficacy of iCBT self-help programs with minimal weekly guidance for people with depression. It aimed to analyze the long-term efficacy of iCBT treatments compared to control conditions as well as long-term efficacy within the iCBT treatment conditions. Additionally, it aimed to conduct subgroup analyses according to the follow-up time points for each outcome. Finally, it examined long-term improvements in quality of life.

**Methods:**

The Cochrane Collaboration Depression, Anxiety, and Neurosis Controlled Trials Register (CCDANCTR), grey literature, reference lists, and correspondence were used to search for published and unpublished randomized controlled trials (RCTs) that reported the long-term or follow-up efficacy of computer-based or iCBT self-help treatments for depression with minimal guidance of up to 10 min/wk. The search took place between 2015 and 2022 (October).

**Results:**

The search resulted in a total of 2809 study abstracts, of which 15 studies (with 17 samples) met all inclusion criteria and were included in the long-term analysis. The results showed that the depression outcomes of all follow-up time points together in the treatment conditions were favored over the control conditions with a medium effect size of 0.43 (n=1689 participants; 9 RCTs; standardized mean difference [SMD] –0.43, 95% CI –0.67 to –0.20; *P*<.001). The analysis of long-term efficacy within the iCBT treatment conditions showed that the follow-up outcomes of the treatment groups were favored over the posttreatment outcomes with a small effect size of 0.20 (n=2196 participants; 17 RCTs; SMD 0.20, 95% CI 0.07-0.49; *P*=.003). Findings for improving quality of life also showed that the iCBT conditions were favored over the control conditions with a small effect size of 0.19 (n=1345 participants; 3 RCTs; SMD 0.19, 95% CI 0.08-0.30; *P*<.001).

**Conclusions:**

This systematic review and meta-analysis found that iCBT self-help interventions had a superior long-term efficacy for individuals with depressive symptoms compared to control groups. The within-group analysis of iCBT treatment conditions also showed statistically significant improvements in reducing depressive symptoms at follow-up compared to posttreatment measurements.

## Introduction

Depression is a worldwide mental disorder and one of the leading causes of disability. According to the World Health Organization (WHO), the incidence of mental disorder conditions increased 13% within the last decade. Approximately 280 million people in the world have depression [1]. COVID-19 Mental Disorders Collaborators [2] estimated that there were approximately 53.2 million additional cases of major depression due to the COVID-19 pandemic across the world. This suggests that the recent pandemic situation increased the urgency for various accessible depression treatments.

Psychotherapy and pharmacotherapy are effective ways to treat depressive disorders [3]. Despite the availability of these evidence-based treatment options, only nearly half of the people with depression receive suitable treatment [4]. Many people with depression are hesitant to take medication or show poor adherence after having taken medication and experiencing side effects [5]. Furthermore, a large amount of individuals with depression do not have the motivation to seek psychotherapy treatment for many reasons, such as perceived stigma, the unavailability of psychotherapists including long waiting lists for the beginning of treatment, probable prohibitive costs, or geographic distance [6,7].

Internet-based self-help programs may be a promising solution for addressing these issues [4]. They offer the people with depression brief and structured therapy with or without any contact with therapists. It can be received at home and is relatively anonymous. It might help avoid stigma and can be used according to the patient’s own schedules and needs.

Moreover, web-based self-help treatments can help the individuals with depression develop usable skills to identify and monitor problematic thoughts and emotions and cope with them [8]. During the internet-based cognitive behavioral therapy (iCBT) self-help treatment, the severity of mild to moderate depressive symptoms may improve, or the waiting period until clinical or face-to-face treatment is available may be bridged.

A growing number of randomized controlled trials (RCTs) [9-13] and meta-analyses [4,14-17] are reporting about the effectiveness of computer-based or iCBT self-help treatments for people with depression.

Furthermore, iCBT self-help treatment can be used as a stand-alone intervention as well as with different levels of support, which can be implemented in different forms, such as brief phone calls, short text messages, emails, or postcards [18]. Several studies are reporting a higher efficacy of guided self-help interventions compared to unguided ones [18-23].

As therapy for depression in general usually requires a long-term rehabilitation process, it is important to study not only the short-term effects of iCBT treatments but also the follow-up or long-term efficacy of this kind of self-help intervention.

Although there are some studies or meta-analyses that studied the effectiveness of iCBT on reducing depressive symptoms at follow-up, the results are inconsistent. Some studies did not find any significant effects of iCBT at follow-up [24,25], whereas other studies [11,26,27] and meta-analyses [14,15] reported significant effects of iCBT treatment over the control group in reducing depression symptoms at follow-up. Andersson et al [28] even reported about a tendency for the guided iCBT group to be superior to group-based cognitive behavioral therapy at 3-year follow-up.

Despite the large amount of studies examining the effectiveness of iCBT self-help treatments at the posttreatment or follow-up stage, there is a lack of meta-analyses analyzing the long-term efficacy of iCBT with a weekly minimal guidance up to 10 minutes during the treatment period.

Therefore, this systematic review and meta-analysis aimed to identify studies that examined the follow-up or long-term efficacy of such iCBT self-help programs with minimal weekly guidance. It aimed to analyze the long-term efficacy of iCBT treatments compared to control conditions as well as the long-term efficacy within the iCBT treatment conditions. Additionally, this review and meta-analysis aimed to conduct subgroup analyses according to the follow-up time points for each outcome. Finally, it examined long-term improvements in quality of life for the participants who are randomized to iCBT self-help interventions compared to control conditions.

## Methods

### Overview

The methods of this long-term meta-analysis refer to an original study published in 2022 [29]; the design and outcomes of posttreatment (short-term) efficacy of iCBT self-help programs for depression with weekly minimal guidance are described there in detail.

The aim of this meta-analysis was to summarize the long-term depression outcomes of the studies that were included in the previous original meta-analysis [29]. We reported the follow-up data, measured at least 8 weeks after posttreatment measurements, and thereby examined the long-term efficacy of iCBT self-help interventions with a weekly minimal guidance (up to 10 minutes) compared to the control conditions of patients who did not receive any treatment before the time point of follow-up measurements. Furthermore, we analyzed the efficacy within the iCBT intervention conditions. Lastly, we analyzed long-term improvements in quality of life for treatment conditions compared to control groups.

### Search Methodology and Study Selection

To identify relevant studies, we searched the Cochrane Collaboration Depression, Anxiety, and Neurosis Controlled Trials Register (CCDANCTR), which contains the searches of MEDLINE (1950 to present), Embase (1974 to present), and PsycINFO (1967 to present); quarterly searches of the Cochrane Central Register of Controlled Trials (CENTRAL); and review-specific searches of additional databases. We also searched international trial registries via the WHO’s trials portal (International Clinical Trials Registry Platform [ICTRP]) and ClinicalTrials.gov to identify unpublished or ongoing studies. We searched sources of grey literature, including dissertations and theses, clinical guidelines, and reports from regulatory agencies (where appropriate). We checked the reference lists of all included studies and relevant systematic reviews to identify additional studies missed from the original electronic searches. We also conducted a cited reference search on the Web of Science.

We did not impose any restriction on date, language, or publication status to the searches.

The selection criteria for studies are shown in Textbox 1.

**Selection criteria.**

**Types of studies**

- Published or unpublished randomized controlled trials, as well as crossover trials

**Diagnosis**

- Studies using one of the following depression questionnaires were accepted: Patient Health Questionnaire (PHQ) [30], Beck Depression Inventory (BDI) [31,32], Hamilton Depression Rating Scale (HDRS) [33], Montgomery Depression Scale (MADRS) [34], The Center for Epidemiologic Studies Depression Scale (CES-D) [35], Hospital Anxiety and Depression Scales (HADS) [36], Kessler Psychological Distress Scale (K-10) [37], Depression Anxiety Stress Scales (DASS) [38], or any other validated depression scale.
- If studies reported more than one type of depression outcome measure, those outcomes were extracted with the highest priority according to the following list: (1) PHQ-9, (2) BDI-II, (3) HDRS, (4) MADRS, (5) CES-D, (6) HADS, and (7) others.

**Types of interventions**

- Studies with experimental internet-based cognitive behavioral therapy (iCBT) self-help programs with weekly minimal guidance (ie, up to 10 minutes) given by a mental health professional or a therapist
- Eligible control comparisons: treatment as usual, waiting list or delayed treatment condition, not active control condition, attention placebo, and psychological placebo

**Types of participants**

- Participants from any racial or ethnic groups aged ≥14 years with depression (ie, measured with a validated depression questionnaire) were included.
- For the long-term analyses, only participants from the intervention conditions that completed the follow-up measurements were eligible, as well as the participants from control conditions that had not received any iCBT self-help program until the follow-up stage.

**Setting**

- Studies conducted in community, primary, secondary, or tertiary services were all eligible for inclusion.

**Types of outcome measures**

- Primary outcome

1. Long-term efficacy of iCBT with weekly minimal guidance (up to 10 minutes): changes in depressive symptomatology at the follow-up stage (treatment group compared with control group, where the participants did not receive any iCBT treatment before the follow-up measurements).

1.1. Subgroup meta-analysis: changes in depression outcomes compared by the time point of follow-up measurements, such as (1) follow-up assessed <6 months after posttreatment measurements, (2) follow-up assessed between 6-8 months after posttreatment measurements, or (3) follow-up assessed >8 months after posttreatment measurements

2. Long-term efficacy within the iCBT treatment conditions: changes of depression symptomatology at follow-up compared with posttreatment outcomes

2.1. Subgroup meta-analysis within the iCBT treatment conditions: changes in depression outcomes analyzed by the time point of follow-up measurements, such as (1) follow-up assessed <4 months after posttreatment measurements, (2) follow-up assessed between 4-7 months after posttreatment measurements, or (3) follow-up assessed >7 months after posttreatment measurements

- Secondary outcome

3. Improvements in quality of life at the follow-up stage, assessed with the use of validated measures

### Data Collection and Analysis

The search took place between 2015 and 2022 (October). The CCDANCTR yielded the abstracts in 2015 and 2018 (to update data). The last update was carried out in 2022. Four independent researchers were involved in the literature search and analysis.

The search resulted in a total of 2809 study abstracts from the CCDANCTR, electronic searches, cross-reference searches, and grey literature. A total of 2756 studies were excluded because they did not meet 1 or more inclusion criteria, and 38 studies could not be included or excluded because of a lack of required information described in the original studies or because there was no publication available to decide on inclusion or exclusion. These study authors were contacted a few times during the process of the meta-analysis. Either there was no response from them, or they could not provide sufficient information for making the decision to exclude or include these studies. Therefore, they are still in the awaiting assessment list. There were also 3 ongoing studies [39-41] that were in process at the time of conducting this meta-analysis.

Finally, 19 studies [6,9,11,27,42-56] met all inclusion criteria, but 4 studies [6,45,51,55] reported only posttreatment measurements and no follow-up measurements. Thus, they could not be included in the long-term analysis.

**Figure 1:**
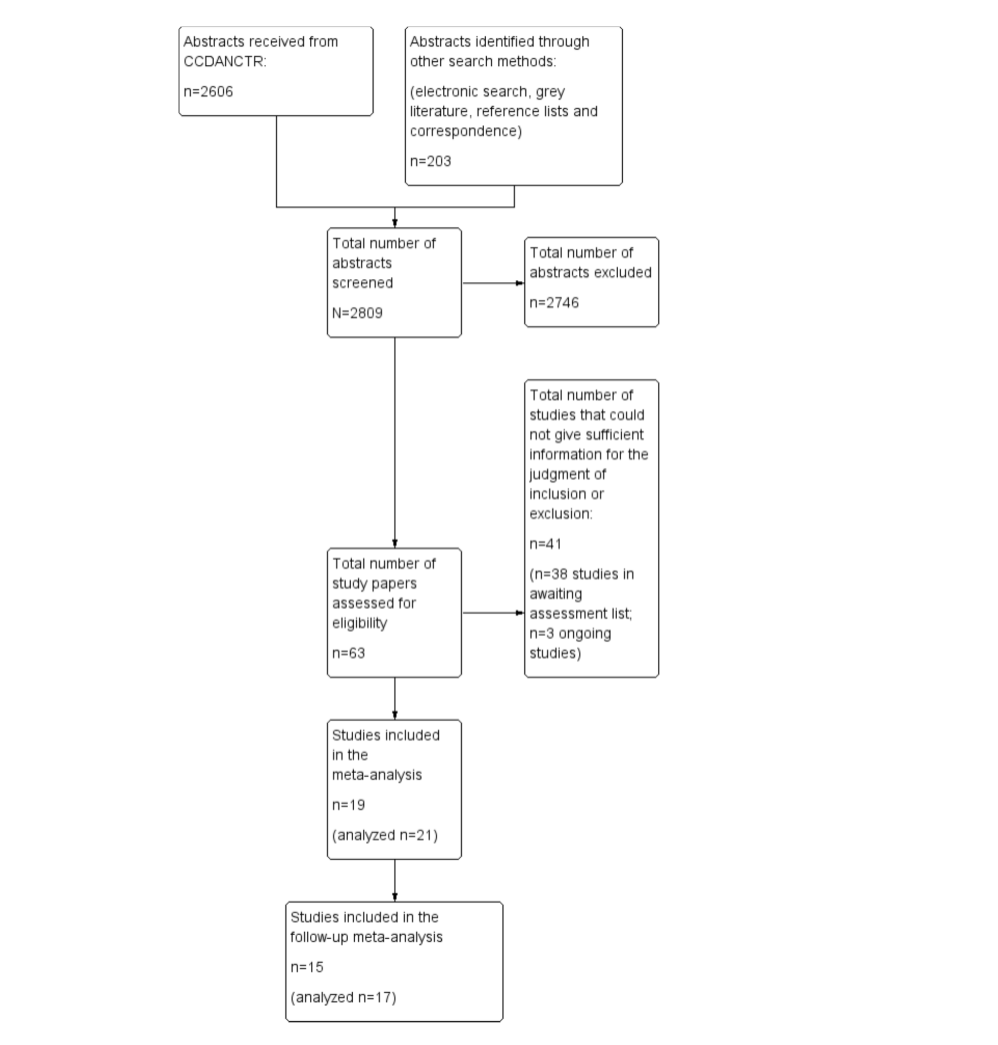
PRISMA (Preferred Reporting Items for Systematic Reviews and Meta-Analyses) flowchart outlining the process of the meta-analysis. CCDANCTR: The Cochrane Depression, Anxiety, and Neurosis Controlled Trials Register.

The results of 2 included studies [42,56] could be used as 2 separate samples due to their 3-arm design. Therefore, in this meta-analysis, a total of 15 studies (with 17 samples) were included. Figure 1 outlines the search process.

We used the latest version of Review Manager (RevMan; version 5.4.1; Cochrane Collaboration) software [57], to extract the characteristics of the included studies, such as the number of participants and means and SDs of outcomes at the posttreatment and follow-up stages.

The quality of the individual studies was assessed with the *Cochrane tool for assessing risk of bias* (Cochrane Collaboration) [58]. The studies were assessed using 7 categories of risk of bias: random sequence generation, allocation concealment, blinding of participants and personnel, blinding of outcome assessment, incomplete outcome data, selective outcome reporting, and other bias. Each category was rated as low, high, or unclear.

### Statistical Analysis

The continuous outcomes such as mean differences (MDs) were pooled into standardized MDs (SMDs) as different questionnaires were used to assess the severity of depression symptomatology in the original studies. We used 95% Cls [58,59].

Furthermore, we tested statistical heterogeneity between studies using a standard chi-square test. We examined the *I*^2^ value using the following overlapping bands provided in the *Cochrane Handbook for Systematic Reviews of Interventions* [58,59]: 0% to 40%=might not be important; 30% to 60%=may represent moderate heterogeneity; 50% to 90%=may represent substantial heterogeneity; and 75% to 100%=may represent considerable heterogeneity.

### Data synthesis

As studies were estimating different treatment effects, we used the random-effects model of meta-analysis.

### Ethical Considerations

All included studies reported having ethics approval. The participants in the original studies provided written informed consent to participate in the studies.

## Results

### Characteristics of the Included Studies

The final research yielded 15 relevant studies (17 samples) [9,11,27,42-44,46-50,52-54,56] for the long-term analysis. The participants in all included studies were randomized in 2 or more groups, where at least one group was an iCBT self-help intervention group with minimal weekly guidance (up to 10 minutes) and the other group was either a control or waitlist group, treatment as usual (TAU), or a not active control condition.

In this long-term meta-analysis, all participants from the intervention conditions who completed the follow-up measurements were included. From the control conditions, only the participants who had not received any treatment until they completed the follow-up measurements were included.

A total of 3226 participants were included in the posttreatment meta-analysis [29]. From this number, for this study, 1280 participants were excluded either from the treatment condition due to not completing the follow-up measurements or from the control condition due to receiving a self-help program after posttreatment measurements. Thus, a total of 1946 participants were included in the long-term meta-analysis.

Measurements of depression symptoms in treatment conditions—as well as in control conditions—were followed up in 9 samples (7 studies) [11,27,42,48,49,52,56]. Another 8 studies [9,43,44,46,47,50,53,54] reported either the follow-up outcomes only for treatment conditions or, if they reported the follow-up outcomes for control conditions, we could not use them due to the participants receiving self-help treatment before the follow-up measurements.

Depression scores were followed up at 12 months after posttreatment measurements only in 2 samples (1 study) [42], and 8-month follow-up depression outcomes were also reported in 1 study [48]. Participants in 4 studies [9,11,27,43] were followed up after 6 months. Two studies [46,50,52] measured follow-up depression scores approximately 4 months after posttreatment measurements. Another 4 studies [44,47,53,54] assessed 3-month follow-up depression scores. Three samples (2 studies) [49,56] measured depression outcomes after 2 or 2.5 months.

Table 1 provides a detailed overview of the follow-up outcomes in each included study, as well as the time point of follow-up measurements (in weeks) and type of control conditions as described in the original studies.

**Table 1:** Detailed characteristics of included studies in the long-term analysis.

Study author, (year)	Follow-up outcomes for the intervention group, mean (SD)	Follow-up outcomes for the control group (without any intervention until follow-up measurements), mean (SD)	Time point of follow-up measurements (weeks)	Number of participants included in the follow-up analysis, n^a^	Follow-up depression outcome	Type of control condition
Andersson et al [43], 2005	13.1 (9.1)	—^b^	24	36	BDI-II^c^	Waitlist (at the follow-up assessment, the control group had completed the internet program)
Berger et al [9], 2011	16.24 (11.4)	—	24	25	BDI-II	Waitlist (at the follow-up assessment, the control group had completed the unguided self-help program)
Choi et al [44], 2012	5.68 (5.39)	—	12	21	PHQ-9^d^	Waitlist (follow-up assessment for the control group was not reported)
Farrer et al [27], 2011	18.4 (10.4)	34.2 (13.5)	24	40	CES-D^e^	TAU^f^
Gilbody et al [42], 2015	8.13 (6.13)	8.45 (6.28)	48	331	PHQ-9	Usual GP^g^ care
Gilbody et al [42], 2015a	7.39 (5.51)	8.45 (6.28)	48	348	PHQ-9	Usual GP care
Klein et al [11], 2016	8.05 (4.20)	9.52 (4.34)	24	636	PHQ-9	TAU alone
Mantani et al [52], 2017	8.92 (6.00)	8.85 (5.93)	17	117	PHQ-9	Switch alone arm
Mohr et al [46], 2013	5.52 (4.45)	—	16	25	PHQ-9	Waitlist (at the follow-up assessment, the control group had completed the coached or self-directed moodManager)
Newby et al [47], 2013	4.05 (3.79)	—	12	40	PHQ-9	Waitlist (participants in the waitlist condition commenced iCBT^h^ immediately after posttreatment assessments)
Newby et al [53], 2017	10.98 (4.49)	—	12	19	PHQ-9	TAU (participants in TAU gained access to the iCBT program after posttreatment measurements)
Proudfoot et al [48], 2004	9.3 (8.5)	14.9 (11.3)	32	186	BDI-II	TAU
Selmi et al [49], 1991	6.17 (5.57)	20.67 (9.89)	8	24	BDI-II	Waitlist (the waitlist control group received the treatment after follow-up measurements)
Smith et al [54], 2017	9.41 (4.71)	—	12	30	PHQ-9	Waitlist (participants in this group had the choice of enrolling in either iCBT or 1 of the 2 self-help books after posttreatment measurements)
Stiles-Shields et al [56], 2019	8.9 (5.88)	11.5 (4.25)	10	20	PHQ-9	Waitlist (the control group began treatment after follow-up measurements)
Stiles-Shields et al [56], 2019a	5.29 (4.46)	11.5 (4.25)	10	18	PHQ-9	Waitlist (the control group began treatment after follow-up measurements)
Titov et al [50], 2010	6.49 (3.94)	—	16	30	PHQ-9	Waitlist (the control group began treatment after the intervention group completed posttreatment assessments)

^a^Total: n=1946.

^b^Not available.

^c^BDI-II: Beck Depression Inventory II.

^d^PHQ-9: Patient Health Questionnaire 9.

^e^CES-D: The Center for Epidemiologic Studies Depression Scale.

^f^TAU: treatment as usual.

^g^GP: general practitioner.

^h^iCBT: internet-based cognitive behavioral therapy.

### Quality of the Included Studies

**Figure 2:**
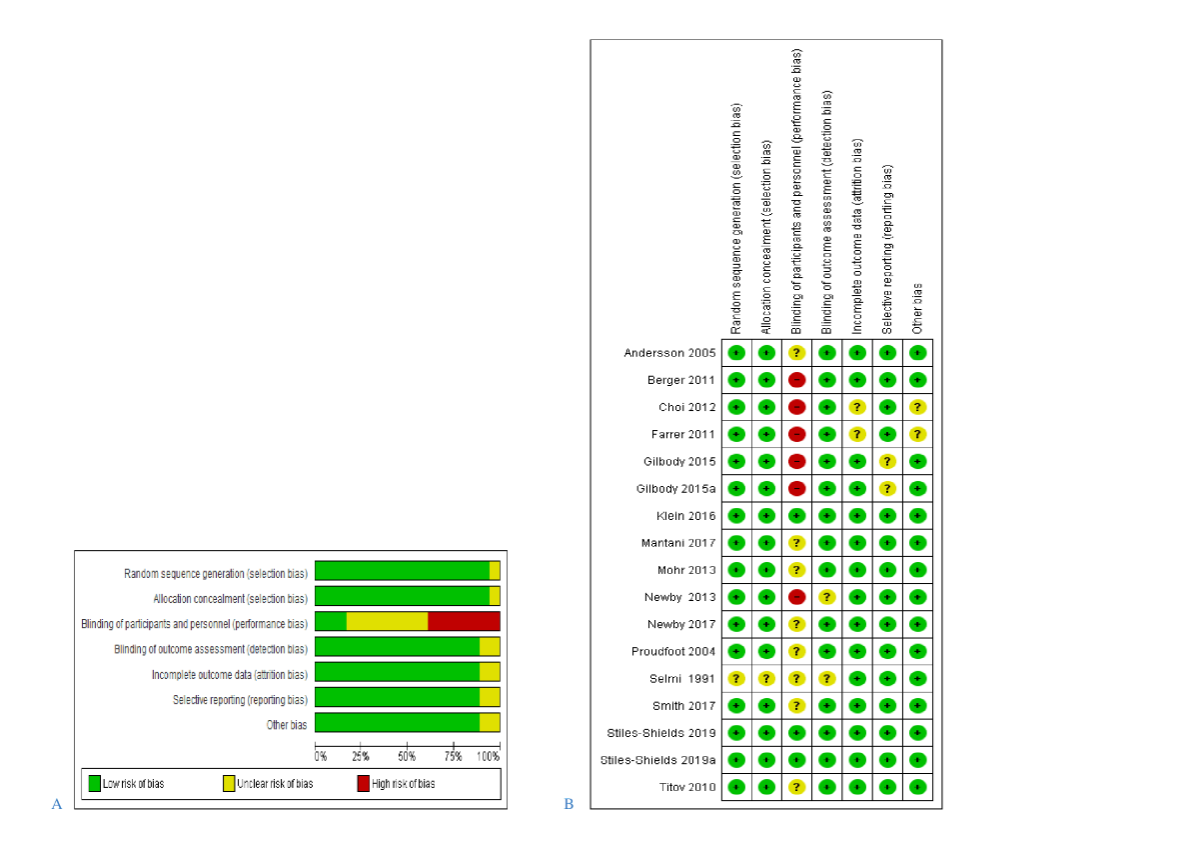
(A) Risk of bias graph: judgements about each risk of bias item presented as percentages. (B) Summary of risk of bias identified for each included study [9,11,27,42-44,46-50,52-54,56].

Figure 2 provides detailed judgments about each risk-of-bias item presented in percentages across all included studies. If there was no sign of bias, it was assessed as “low risk of bias.” If the original study authors did not report sufficient information to judge existing bias, it was assessed as “unclear.” If we had suspicion of real existing bias, it was assessed as “high risk of bias.”

The participants of all included studies were randomized, and all studies except one [49] described their randomization method in detail. The process of random allocation sequence was circumstantially described in all of the included studies except one [49]. Therefore, there was a low risk of selection bias.

If participants as well as personnel were blinded, the risk of performance bias was assessed as low. If personnel were not blinded, it was assessed as high risk. If there was not sufficient information about the blinding of personnel, performance bias was assessed as unclear. In most of the included studies except 2 studies (3 samples) [11,56], this kind of bias was assessed as high or unclear.

There was a low risk of detection bias in most of the included studies except 2 studies [47,49]. In these 2 studies, there was not enough information provided to permit judgment about the blinding of outcome assessment.

Two studies [27,44] did not report sufficient information to judge the risk of attrition bias. The remaining studies had a low risk of incomplete outcome data.

All included studies except 1 study (2 samples) [42] reported all predefined outcomes. Therefore, there was a low risk of reporting bias.

Finally, there was no sign of high risk of other sources of bias. Two studies [27,44] did not report sufficient information about other bias.

In total, the risk of bias of all included studies could be assessed as “low to moderate,” except for performance bias, which could be assessed as “moderate to high” due to the lack of blinding of participants and personnel in the original studies.

### Test of Heterogeneity

We chose the random-effects model to interpret the results of the long-term meta-analysis. The heterogeneity of the effect size samples was automatically tested in RevMan with *I*^2^ values for the first primary outcome.

The results of the heterogeneity test for iCBT treatment efficacy at the follow-up time points showed substantial or considerable heterogeneity (*I*^2^=75%; *P*<.001).

### Primary Outcomes

#### 1. Long-Term Efficacy (iCBT Compared to Control Condition)

A total of 7 studies [11,27,42,48,49,52,56] assessed the follow-up outcomes in both conditions: for iCBT interventions as well as for control conditions. For 2 studies [42,56], 2 separate samples were usable; therefore, 9 samples were analyzed with a total 1689 participants using follow-up end point scores of depression symptoms.

The outcomes of long-term efficacy of iCBT self-help programs were assessed in various depression scales: Patient Health Questionnaire 9 (PHQ-9) [30], Beck Depression Inventory II (BDI-II) [31], and The Center for Epidemiologic Studies Depression Scale (CES-D) [35]. Therefore, we had to pool MDs into SMDs.

**Figure 3:**
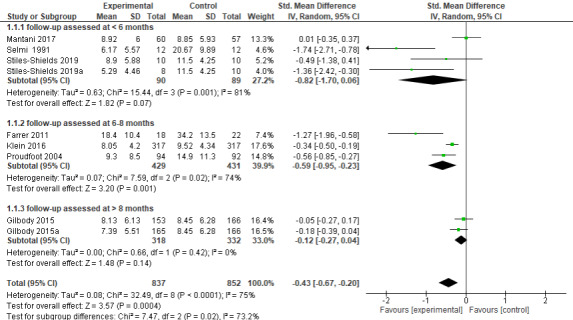
Forest plot of standardized mean difference (95% CI) in change of depressive symptoms for intervention and control conditions at follow-up [11,27,42,48,49,52,56].

The long-term analysis of all depression scales and all follow-up time points together showed statistically significant differences between iCBT self-help treatment groups and control conditions that included participants who did not receive any treatment until follow-up measurements. Namely, the follow-up outcomes in the treatment conditions were favored over the control conditions with a medium effect size of 0.43 (n=1689 participants; 9 RCTs; SMD –0.43, 95% CI –0.67 to –0.20; *Z*=3.57, *P*<.001; *I*^2^=75%, *P*<.001; see Figure 3).

#### 1.1. Subgroup Analysis (iCBT Compared to Control Condition)

The analysis of depression outcomes, using the subgroups of different follow-up stages (followed up at <6 months, 6-8 months, or >8 months), showed that the iCBT conditions were favored over the control conditions.

The participants in the iCBT treatment groups who were followed up <6 months after posttreatment measurements improved their depression symptoms with a large effect size of 0.82. However, this improvement was not statistically significant (n=179 participants; 4 RCTs; SMD –0.82, 95% CI –1.70 to 0.06; *Z*=1.82, *P*=.07; *I*^2^=81%, *P*<.001; see Figure 3).

The depression outcomes followed up between 6-8 months after treatment also showed statistically significant improvements in depression symptoms in iCBT self-help groups with a moderate effect size of 0.59 (n=860 participants; 3 RCTs; SMD –0.59, 95% CI –0.95 to –0.23; *Z*=3.20, *P*=.001; *I*^2^=74%, *P*=.02; see Figure 3).

The analysis of 2 samples, where the follow-up was assessed >8 months after posttreatment measurements, showed that the iCBT intervention conditions were favored over the control conditions in improving depression symptoms with a small effect of 0.12. However, this improvement was not statistically significant (n=650 participants; 2 RCTs; SMD 0.12, 95% CI –0.27 to 0.04; *Z*=1.48, *P*=.14; *I*^2^=0%, *P*=.42; see Figure 3).

#### 2. Long-Term Efficacy Within iCBT Treatment Conditions

The depressive symptoms of the participants in the iCBT self-help intervention groups were followed up in 17 samples (15 studies) [9,11,27,42-44,46-50,52-54,56].

Depression outcomes were assessed in an iCBT treatment condition among 1133 participants at the posttreatment stage. A total of 1063 (95.5%) out of 1113 participants in the intervention groups completed the follow-up measurements.

**Figure 4:**
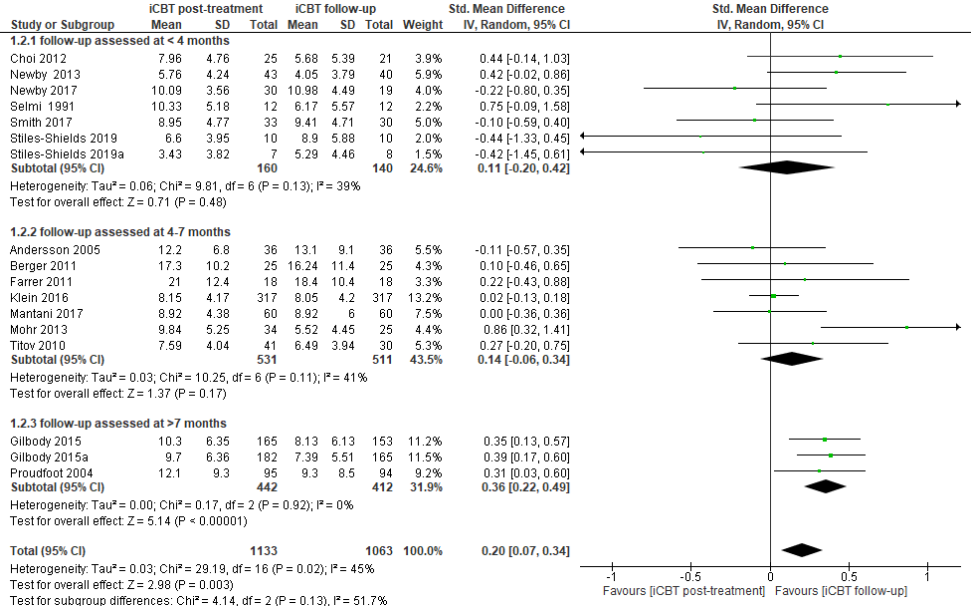
Forest plot of standardized mean difference (95% CI) in change of depressive symptoms for intervention conditions: posttreatment stage compared to follow-up stage [9,11,27,42-44,46-50,52-54,56]. iCBT: internet-based cognitive behavioral therapy.

The results of this comparison between the depression outcomes at the posttreatment stage and follow-up stage showed statistically significant differences between these 2 time points. Namely, the participants in the iCBT self-help intervention groups continued to improve their depressive symptoms even a few months after they received self-help programs with minimal guidance. Specifically, the follow-up outcomes of the treatment groups were favored over the posttreatment outcomes with a small effect size of 0.20 (n=2196 participants; 17 RCTs; SMD 0.20, 95% CI 0.07-0.49; *Z*=2.98, *P*=.003; *I*^2^=45%, *P*=.02; see Figure 4).

#### 2.1. Subgroup Analysis of iCBT Treatment Conditions

Moreover, we analyzed the depression outcomes by the time point of follow-up measurements, such as (1) follow-up assessed <4 months after posttreatment measurements, (2) follow-up assessed between 4-7 months after posttreatment measurements, or (3) follow-up assessed >7 months after posttreatment measurements.

The subgroup analysis showed improvements of depressive symptoms in the iCBT intervention groups that were followed up in <4 months after posttreatment measurements with a small effect size of 0.11. However, these changes were not statistically significant (n=300 participants; 7 RCTs; SMD 0.11, 95% CI –0.20 to 0.42; *Z*=0.71, *P*=.48; *I*^2^=39%, *P*=.13; see Figure 4).

The participants of the iCBT intervention groups that were followed up between 4-7 months after posttreatment measurements also showed improvements in depressive symptoms at the follow-up stage with a small effect size of 0.14. However, these changes were not statistically significant (n=1042 participants; 7 RCTs; SMD 0.14, 95% CI –0.06 to 0.34; *Z*=1.37, *P*=.17; *I*^2^=41%, *P*=.11; see Figure 4).

The analysis of the intervention groups where the participants were followed up >7 months after posttreatment measurements showed statistically significant improvements in depressive symptoms with a small effect size of 0.36 (n=854 participants; 3 RCTs; SMD 0.36, 95% CI 0.22-0.49; *Z*=5.14, *P*<.001; *I*^2^=0%, *P*=.92; see Figure 4).

### Secondary Outcome: 3. Improvements in Quality of Life at Follow-Up

A total of 3 samples (2 studies) [11,42] assessed improvements in quality of life at follow-up among 1345 participants in the intervention and control conditions together. Klein et al [11] used the Mental Composite Score of SF-12 [60] to assess improvements in quality of life. Gilbody et al [42] assessed the quality of life with the SF-36 [61]. Low scores in this outcome correspond to low improvements in quality of life.

The results showed statistically significant improvements for both follow-up time points: (1) follow-up assessed 6 months after posttreatment measurements and (2) follow-up assessed 12 months after posttreatment measurements.

**Figure 5:**
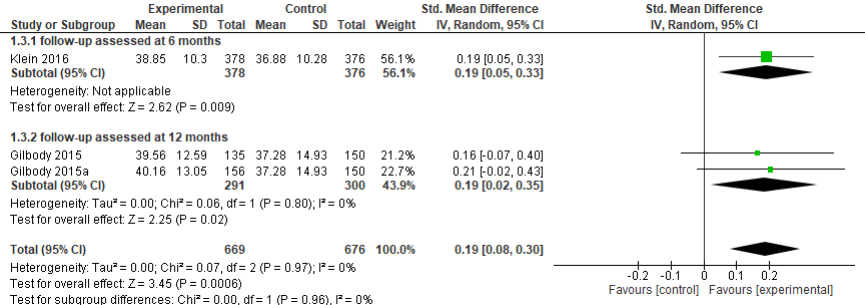
Forest plot of standardized mean difference (95% CI) in quality of life (low=poor) for intervention and control conditions at follow-up [11,42].

Namely, the improvement in quality of life among the participants in the iCBT conditions were favored over the participants in the control conditions with a small effect size of 0.19, which is statistically significant (n=1345 participants; 3 RCTs; SMD 0.19, 95% CI 0.08-0.30; *Z*=3.45, *P*<.001; *I*^2^=0%, *P*=.97; see Figure 5).

## Discussion

### Principal Findings

As depression therapy is a long-term rehabilitation process, it is important to examine not only short-term effects of iCBT self-help treatments but also the follow-up or long-term efficacy of this kind of intervention.

This systematic review and meta-analysis identified 17 samples (15 studies) that assessed the long-term efficacy of iCBT self-help interventions with minimal guidance (up to 10 min/wk) for depression at follow-up among 1946 participants.

### Long-Term Efficacy of iCBT Compared to Control Condition

The analysis of long-term efficacy in 9 samples (among 1689 participants) revealed that the efficacy of iCBT interventions with a weekly minimal guidance showed statistically significant improvements in reducing depressive symptoms (with a medium effect size of 0.43) compared to the control conditions, where the participants did not receive any treatment until the follow-up measurements. This finding is well supported by a current meta-analysis, where Karyotaki et al [14] reported that guided iCBT self-help interventions reduced depressive symptoms compared with TAU at the 6-month follow-up. Zhou et al [62] reported similarly about the positive effect of iCBT interventions on reducing depression levels, which was significant at the <3-month follow-up.

We also conducted a subgroup analysis for this outcome according to the time point of follow-up measurements and found that the iCBT intervention groups were favored over the control condition in reducing depressive symptoms at every stage of follow-up (at <6 months, 6-8 months, or >8 months), although these improvements were statistically significant only at the stage of 6- to 8-month follow-up. Hence, the results showed the inconsistency of the significance level of the efficacy of iCBT in reducing depressive symptoms on the different stages of follow-up. These findings are supported by the findings of Zhou et al [62]. In addition, the results of this outcome indicate a high degree of heterogeneity (*I*^2^=75%).

These findings suggest that iCBT self-help interventions for depression with minimal weekly guidance (up to 10 minutes) can be useful in reducing depressive symptoms not only at the posttreatment stage but also at the follow-up stage.

### Long-Term Efficacy Within iCBT Treatment Conditions

A total of 17 samples (15 studies) with 1133 participants at the posttreatment time point and 1063 participants at the follow-up time point were included in the analysis of efficacy within the iCBT treatment conditions at follow-up.

Our previous meta-analysis [29] reported statistically significant improvements of depressive symptoms in iCBT treatment conditions compared to control conditions at the posttreatment stage. The long-term analysis of iCBT treatment conditions revealed that the participants who received iCBT self-help with weekly minimal guidance improved their depressive symptoms with a statistically significant, small effect size of 0.20 for all follow-up time points together compared to the outcomes at the posttreatment time point. This result is well supported by the findings of a previous meta-analysis [62] that reported about significant within-group effects of iCBT interventions on depression improvements at 3-month follow-up.

A subgroup analysis for this outcome according to the time point of follow-up measurements (ie, at <4 months, 4-7 months, or >7 months) within iCBT intervention groups also showed improvements in reducing depressive symptoms at every stage of follow-up. However, these improvements were statistically significant only at the follow-up time point of >7 months after posttreatment measurements.

### Improvements in Quality of Life at Follow-Up

As the secondary outcome, we analyzed long-term improvements in quality of life within 3 samples with a total of 1345 participants in the iCBT intervention and control groups together.

The analysis showed small but statistically significant improvements in quality of life at the follow-up stage (at 6 and 12 months) in the participants of the intervention groups who received iCBT self-help treatments with weekly minimal guidance. This result approximates the findings of the recent meta-analysis by Han and Kim [15], who reported a small effect of internet-based intervention on improving quality of life compared to control groups at follow-up.

### Strengths, Limitations, and Implications

Among the strength of this systematic meta-analysis is the clearly defined set of inclusion and exclusion criteria regarding participants, intervention, study design, outcomes, etc. [63].

Moreover, we examined the funnel plots for each outcome to assess the likely presence of publication bias. There was no evidence of possible asymmetry for either outcome.

In addition, we were able to conduct subgroup analyses and examined the efficacy of iCBT with minimal guidance at different stages of follow-up measurements.

Finally, the quality of the included studies was rather high, which allowed us to conclude that this meta-analysis is relatively free from critical bias. There was overall low risk of bias for all included studies. The quality of only 1 case—performance bias—was assessed as moderate to high. However, it is very difficult or sometimes even impossible to achieve total blinding of personnel and participants in such psychotherapeutic studies with minimal guidance.

This systematic review and meta-analysis has also several limitations that should be taken into consideration when interpreting the results.

First, one of the important limitations was the lack of follow-up data reported in the original studies. Only 9 out of 17 included samples assessed follow-up outcomes in both conditions: for iCBT interventions as well as for control conditions. Furthermore, relatively few studies examined improvements in quality of life.

Second, in the included studies, various iCBT programs with different number of sessions were used, which may report different effect sizes and can be a source of high heterogeneity between the included studies. Additionally, the inclusion of studies with a different types of control conditions (eg, waitlist, TAU, or usual general practitioner care), as well as the inclusion and comparation of studies with a different level of technological development (eg, an iCBT self-help program in 1991 compared to iCBT-based multimedia in 2019), could make it hard to interpret the results.

Furthermore, the different time points of follow-up measurements in the included studies may have a role in analyzing the long-term efficacy of iCBT with minimal guidance. Nonetheless, subgroup analysis was carried out to examine these differences.

Finally, this meta-analysis included only published outcomes of follow-up measurements. The potential for studies reporting small or null findings at the follow-up stage and not being published through either reluctance from authors or journal editors dismissing them may be a problem. Publication bias is, however, a problem for all researchers and not only for this meta-analysis.

### Conclusions

In conclusion, this systematic review and meta-analysis found that iCBT self-help interventions with weekly minimal guidance of up to 10 minutes had superior long-term efficacy for individuals with depressive symptoms compared to control groups.

The within-group analysis of iCBT treatment conditions showed statistically significant improvements in reducing depressive symptoms at the follow-up stage compared to posttreatment measurements.

In addition, the analysis of improvements in quality of life at follow-up (at 6 and 12 months) showed statistically significant improvements in the participants that received iCBT self-help treatments compared to the control conditions.

However, the statistical significance of the long-term effectiveness of iCBT self-help programs for depression at various follow-up stages was inconsistent. Furthermore, it is important that future studies systematically examine the moderator factors at follow-up for this inconsistency, such as the number of previous depression episodes, severity of depression, symptom duration, etc.

Moreover, further research should be undertaken to develop practicable approaches to include iCBT interventions in health care systems, as it would help patients with mild to moderate depressive symptoms in reducing the severity of their depressive symptoms or to bridge the waiting period until they receive clinical or face-to-face treatment.

## Abbreviations

BDI: Beck Depression Inventory
CCDANCTR: The Cochrane Depression, Anxiety, and Neurosis Controlled Trials Register
CENTRAL: Cochrane Central Register of Controlled Trials
CES-D: The Center for Epidemiologic Studies Depression Scale
iCBT: internet-based cognitive behavioral therapy
ICTRP: International Clinical Trials Registry Platform
MD: mean difference
PHQ: Patient Health Questionnaire
RCT: randomized controlled trial
RevMan: Review Manager
SMD: standardized mean difference
TAU: treatment as usual
WHO: World Health Organization

## Data Availability

The data sets used and analyzed during this review and meta-analysis are available from the corresponding author upon reasonable request.
